# Unique Case of Congenital Duodenal Atresia and a Choledochal Cyst and the Hypothesis of Their Embryological Evolution

**DOI:** 10.3390/children7080099

**Published:** 2020-08-18

**Authors:** Brittany Downing, Mohammad Y. Bader, Frank P. Morello, Ranjit I. Kylat

**Affiliations:** 1 Department of Pediatrics, College of Medicine, University of Arizona, Tucson, AZ 85719, USA; btalamantes@peds.arizona.edu (B.D.); mbader@email.arizona.edu (M.Y.B.); 2 Banner University Medical Center, Tucson, AZ 85719, USA; fmorello@radiology.arizona.edu; 3 Department of Medical Imaging, College of Medicine, University of Arizona, Tucson, AZ 85719, USA

**Keywords:** choledochal cyst, duodenal atresia, cholecystectomy, embryology, metaplasia

## Abstract

The concomitant occurrence of duodenal atresia (DA) and a choledochal cyst (CC) has rarely been reported. Knowledge of both the presentation and management of this rare co-occurrence is imperative in avoiding potential complications and sequelae, such as biliary metaplasia. Herein we describe a female infant born at 32 weeks gestational age who was diagnosed with duodenal atresia and annular pancreas postnatally, who had subsequent findings of malrotation and a choledochal cyst, as seen from contrast imaging. Uncomplicated repair of the DA and obstruction was performed at 4 days of life. She re-presented 2 years later with non-bloody, nonbilious emesis and was found to have elevated amylase, lipase and liver enzymes. Imaging revealed dilated intra-hepatic ducts, a distended gallbladder and a large choledochal cyst. She underwent a cholecystostomy tube placement followed by a definitive choledochal cyst excision with immediate improvement following surgery and full resolution of symptoms before discharge.

## 1. Introduction

Congenital obstruction of the duodenum can be complete or partial and due to an intrinsic or extrinsic etiology. Intrinsic obstruction is often caused by duodenal atresia (DA) and occurs in approximately 1 in 5000 to 1 in 10,000 live births [1]. Extrinsic obstruction can be caused by intestinal malrotation, annular pancreas, gastro-duodenal duplication anomalies and pseudocysts of the pancreas and biliary tree. The incidence of congenital choledochal cysts (CC) is reported as 1 in 15,000 live births [2]. The concomitant occurrence of a congenital choledochal cyst with duodenal atresia is very rare, with only 12 cases reported. We report a case of concomitant CC with DA in a preterm 32-week gestational age infant and postulate a hypothesis for its embryological evolution.

## 2. Case Summary

**Figure 1:**
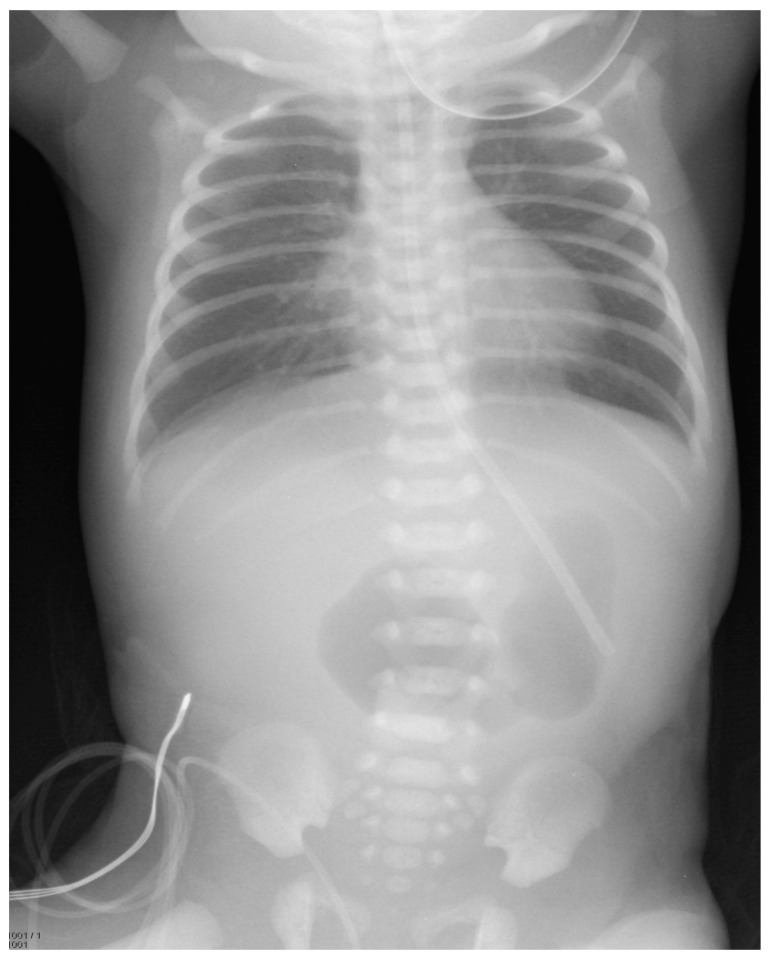
Plain abdominal radiograph taken soon after birth, showing a double-bubble in the lower abdomen.

**Figure 2:**
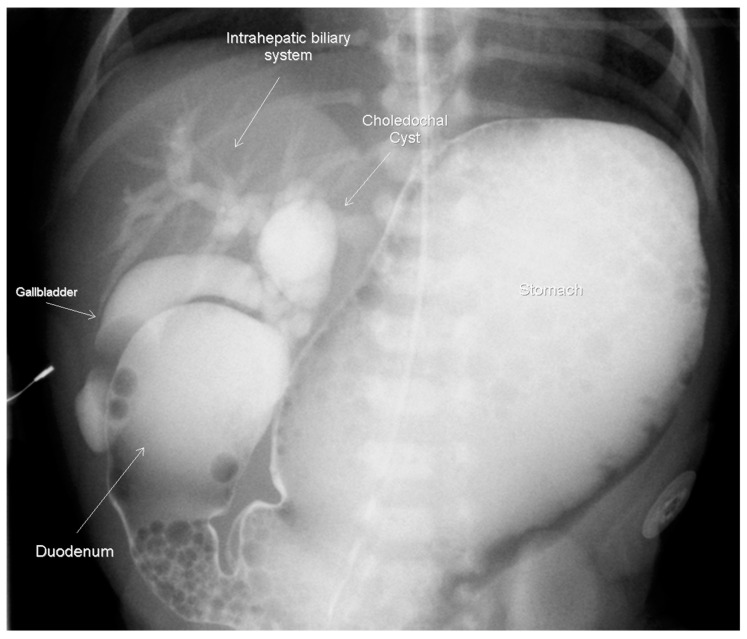
Upper GI contrast study on day-of-life 2, demonstrating the duodenal obstruction with reflux into the dilated biliary tree and a choledochal cyst approximately 14 × 10 mm.

**Figure 3:**
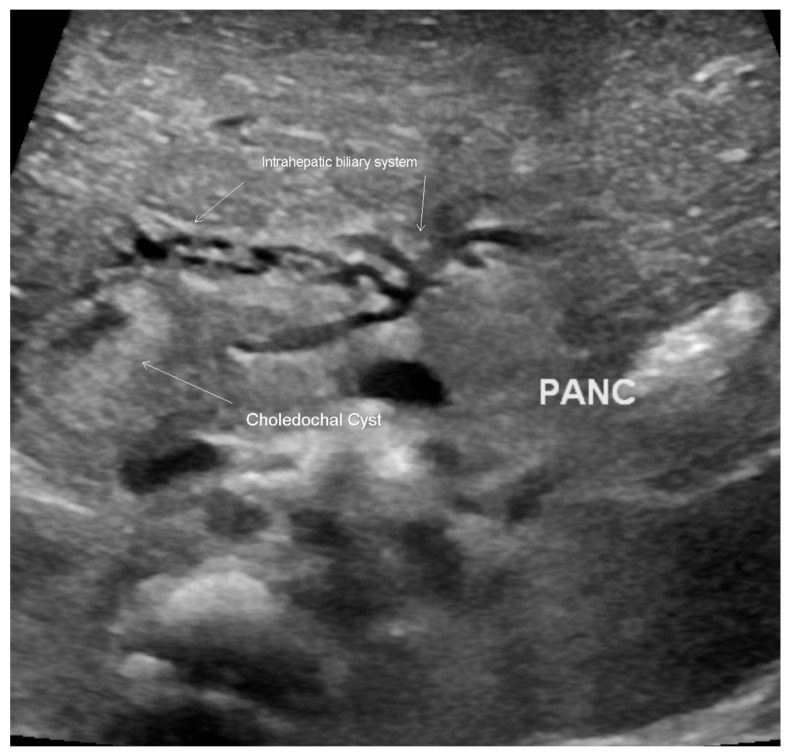
Ultrasound scan of the abdomen, revealing a choledochal cyst with dilatation of the intrahepatic biliary system.

A female infant weighing 1700 g was born at 32 weeks gestational age to a 42-year-old mother. The pregnancy was complicated by gestational diabetes, with a non-immune rubella titer and prenatal ultrasound (US) revealing polyhydramnios and an echogenic bowel. Imaging after birth revealed a double-bubble sign typical for duodenal atresia. (Figure 1) A subsequent upper gastrointestinal contrast study revealed obstruction in the first part of the duodenum, malrotation, and a choledochal cyst. (Figure 2) A follow-up abdominal ultrasound (US) showed reversal of the expected superior mesenteric artery and superior mesenteric vein with no swirling of the vascular pedicle, as expected in the setting of intestinal malrotation. The infant underwent an uncomplicated DA repair and Ladd’s procedure at 4 days of life. Of note, an annular pancreas was visualized intra-operatively. Portal structures were not explored at this time in favor of repeat imaging of the biliary tree when the infant was older. The postoperative course was significant for elevated direct bilirubin levels, presumably secondary to cholestasis due to prolonged parenteral nutrition. Abdominal US, on postoperative day 14, revealed a persistent, focally dilated bile duct and extrahepatic biliary ducts without the associated symptoms. (Figure 3 Bilirubin normalized and the patient was discharged at a corrected gestational age of 38 weeks with adequate oral intake and weight gain.

**Figure 4:**
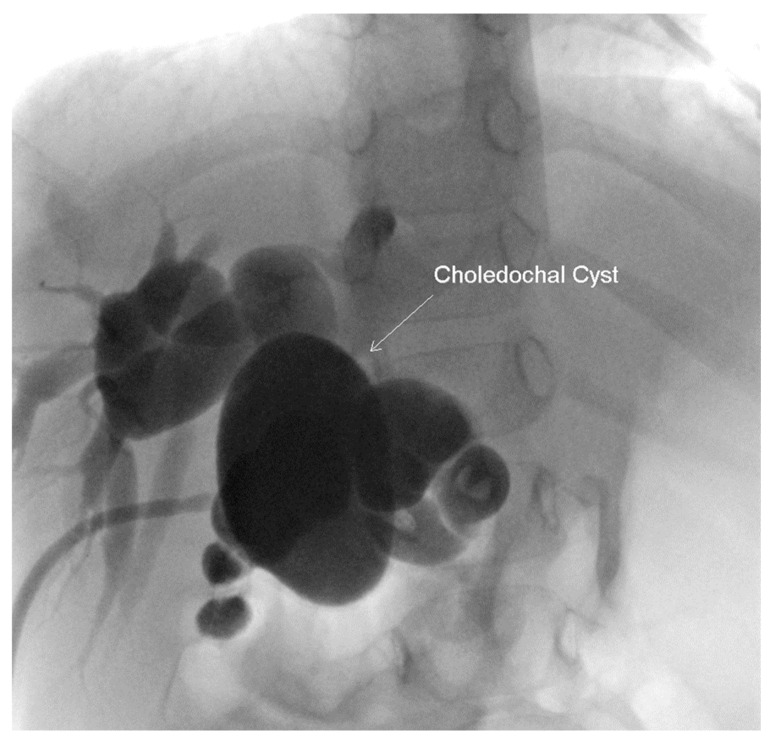
Intra-operative cholangiogram performed at two years of age, demonstrating a persistently dilated biliary tree with a revisualization of the choledochal cyst approximately 59 × 37 mm.

At two years of age the patient presented to the emergency department with feeding intolerance, persistent emesis, elevated liver transaminases (AST 244, ALT 288), elevated amylase >1446 U/L and lipase 465 IU/L. Magnetic resonance imaging (MRI) revealed significantly dilated intra- and extra-hepatic ducts, a distended gallbladder and a large choledochal cyst. The physical exam was significant for ascites and jaundice. She underwent an urgent laparotomy, which found bilious ascites requiring subsequent placement of a cholecystostomy tube. A cholangiogram was then performed due to the persistent feeding intolerance. This re-demonstrated the intra- and extra-hepatic ductal dilation and incomplete biliary obstruction. (Figure 4) She underwent a cholecystectomy, choledochal cyst excision and hepatico-duodenostomy, which she tolerated well. By post-operative day 7 the patient demonstrated substantial clinical improvement and was deemed appropriate for discharge. On a follow-up examination at 3 years of age, the patient had remained asymptomatic with normal growth and liver function.

## 3. Discussion

This case demonstrates a case of antenatally formed DA and CC with concomitant anomalies requiring surgical intervention due to an obstructed hepatobiliary system 2 years after the initial findings. The embryologic origin of the duodenal atresia centers on the failure of the recanalization of the duodenum after the 7th week of gestation and can be associated with other congenital anomalies such as Down’s syndrome, which is present in 25–40% of cases [2,3]. Other associated anomalies include VACTERL (vertebral, imperforate anus, cardiac, tracheo-esophageal fistula, renal anomalies and limb abnormalities), malrotation, annular pancreas, biliary tract abnormalities as well as cardiac and mandibulofacial anomalies. Two of these were described in our patient case [4]. Though uncommon, the etiology of both DA and CC occurring during the congenital period and their embryologic association have been largely debated. One such theory by Boyden et al. proposes that a “local traffic jam” occurs between the duodenum and the entry of the hepatopancreatic and accessory pancreatic ducts during the 7th week of embryogenesis [4]. This “traffic jam” hinders the recanalization of the duodenum and leads to a pancreaticobiliary maljunction (PBM, the congenital fusion of pancreatic and biliary ducts outside the Ampulla of Vater) [4]. In 1999, Ando et al. described PBM as the link between the development of a CC and DA. His own description of a PBM involved an abnormal fusion of the right ventral pancreatic duct with the bile/pancreatic ducts during embryogenesis leading to “an oddly shaped union that could present at birth with or without bile duct dilatation” [3].

Similarly, pediatric cases reported in the literature since the early 1990s have included findings of PBM associated with duodenal obstruction and subsequent choledochal cysts. The first case of CC and its surgical removal was reported by Bailey et al. in 1993 [5]. Iwai et al. followed, reporting 9 cases of CC-associated duodenal obstruction [6]. Iwai’s case review describes 9 patients with annular pancreas and either duodenal stenosis or atresia. He reported that each patient required surgical intervention a minimum of two years after the findings, due to the symptomatic development of choledochal cysts [6]. More recently, a case report describing a 35-week gestational age male born with DA post duodenoduodenostomy, which required surgical intervention at 2 months of age due to a dilated common bile duct whose pathology was consistent with a Type I CC [7]. In his case report, Shih et al. noted an increased presence of annular pancreas in their review of 6 reported cases of duodenal atresia or stenosis and choledochal cysts. This suggests an increased risk of duodenal obstruction and PBM is present with embryologic events involving the ventral pancreas rotation, leading one to support the association between annular pancreas and choledochal cyst formation [8].

This same finding was described by Iwai et al. involving a 37-week gestational age female infant born with DA repaired at birth, who presented at 4 years of age with jaundice, vomiting and was found to have a choledochal cyst and dilatation of the left intrahepatic duct requiring surgical resection [6]. Iwai theorized the reason that no choledochal cysts were identified at the initial duodenal repair was secondary to only minimal common bile duct dilatation with a gradual increase in size over time after surgery. Sugimoto et al. similarly suggests that the common bile duct in their case, became gradually dilated after DA repair because of a “congenital pancreaticobiliary anomaly” [9]. Our patient was found to have a choledochal cyst during the initial upper GI study on day-of-life 2, implying a congenital presence. The asymptomatic nature of our patient until years later, may suggest a possible secondary insult leading to its later presentation or a gradual dilatation of the bile duct after duodenal repair as hypothesized above.

Despite the rarity of this association, the proposed mechanisms have all seemingly involved an anomalous formation of the pancreaticobiliary junction. Early case reports have nearly identical imaging findings, including an anomalous pancreaticobiliary ductal junction found during the intraoperative cholangiogram, annular pancreas and CC [8,9,10]. The later presentation of patients described in previous cases and findings of intrahepatic and biliary duct dilatation in the setting of duodenal repair years earlier parallels our case and supports the hypothesized claims of gradual symptomatic CBD dilatation secondary to a congenital presence of PBM.

## 4. Conclusions

The simultaneous occurrence of congenital duodenal atresia and a choledochal cyst, although rare, have common anatomical findings in all case reports throughout the years. These findings offer an underlying connection between an anomalous arrangement of the pancreaticobiliary system, duodenal obstruction, annular pancreas and the gradual dilation of bile ducts into symptomatic choledochal cysts. This emphasizes the importance of early evaluation in infants presenting with DA, to identify abnormalities such as CC and annular pancreas to prevent the development of dangerous sequelae, such as chronic cholangitis, pancreatitis, biliary metaplasia or the need for invasive surgical interventions in later childhood.

## 5. Financial Disclosures

No financial relationships relevant to this article to disclose.

## References

[1] Desoky S., Kylat R.I., Udayasankar U.K., Gilbertson-Dahdal D. (2018). Managing neonatal bowel obstruction: clinical perspectives. Res. Rep. Neonatol..

[2] Morris G., Kennedy A., Cochran W. (2016). Small Bowel Congenital Anomalies: A Review and Update. Curr. Gastroenterol. Rep..

[3] Boyden E.A., Cope J.G., Bill A.H. (1967). Anatomy and embryology of congenital intrinsic obstruction of the duodenum. Am. J. Surg..

[4] Ando H., Kaneko K., Ito F., Seo T., Harada T., Watanabe Y. (1999). Embryogenesis of pancreaticobiliary maljunction inferred from development of duodenal atresia. J. Hepatobiliary Pancreat. Surg..

[5] Bailey P.V., Tracy T.F., Connors R.H., Mooney D.P., Lewis J.E., Weber T.R. (1993). Congenital duodenal obstruction: A 32-year review. J. Pediatr. Surg..

[6] Iwai A., Hamada Y., Takada K., Inagaki N., Nakatake R., Yanai H., Miki H., Araki Y., Sato M., Ono S. (2009). Choledochal cyst associated with duodenal atresia: case report and review of the literature. Pediatr. Surg. Int..

[7] Goh M.F.J., Mak M.H.W., Low Y., Ong C.C.P. (2019). Congenital or acquired? Obstructive jaundice in reoperated duodenal atresia. BMJ Case Rep..

[8] Shih H.S., Ko S.F., Chaung J.H. (2005). Is there an association between duodenal atresia and choledochal cyst?. J. Pediatr. Gastroenterol. Nutr..

[9] Sugimoto T., Yamagiwa I., Obata K., Ouchi T., Takahashi R., Suzuki R., Shimazaki Y. (2004). Choledochal cyst and duodenal atresia: A rare combination of malformations. Pediatr. Surg. Int..

[10] Komuro H., Makino S., Tahara K. (2000). Choledochal cyst associated with duodenal obstruction. J. Pediatr. Surg..

